# Apocrine tubular adenoma on the palm: A case report

**DOI:** 10.1097/MD.0000000000028002

**Published:** 2021-12-10

**Authors:** Jangyoun Choi, Jeong Hwa Seo, Jong Yun Choi, Bommie Florence Seo, Ho Kwon, Sung-No Jung

**Affiliations:** a Department of Plastic and Reconstructive Surgery, Seoul St. Mary's Hospital, College of Medicine, The Catholic University of Korea, Seoul, South Korea; b Department of Plastic and Reconstructive Surgery, Uijeongbu St. Mary's Hospital, College of Medicine, The Catholic University of Korea, Gyeonggi, South Korea.

**Keywords:** adnexal and skin appendage, neoplasms, tubular sweat gland adenomas

## Abstract

**Rationale:**

Tubular apocrine adenoma (TAA) is a very rare benign neoplasm of the apocrine gland. The typical anatomical locations are mostly hair-bearing areas, where normal apocrine glands are abundant.

**Patient concerns:**

We report the case of a 40-year-old patient with a tubular apocrine adenoma on a non-hair-bearing area.

**Diagnoses:**

Ultrasonography showed a 0.4-cm-sized hypoechoic nodule in the subcutaneous plane of the left palm at the fourth carpometacarpal joint level.

**Interventions:**

Surgical resection was performed and histopathological examination revealed lobules of well-differentiated dilated tubular structures at the dermis level.

**Outcomes:**

At 1 year of postoperative follow-up, the patient was completely recovered without signs of recurrence.

**Lessons:**

Diagnosis of TAA can be misleading due to its unusual location and inconspicuous appearance. Immunohistochemistry for epithelial membrane antigen and gross cystic disease fluid protein-15 is crucial for the proper diagnosis. Complete excision is recommended to prevent recurrence.

## 1 Introduction

Tubular apocrine adenoma (TAA) is a very rare benign neoplasm of the apocrine gland. Most commonly found in the scalp, other areas of occurrence have been described, including the face, axilla, and genitalia.^[1]^ The typical anatomical locations are mostly hair-bearing areas, where normal apocrine glands are abundant. On gross examination, it is nodular, slow-growing, and fixed to the dermis. Histologically, TAA is characterized by tubular structures located in the dermis. The tubules are composed of inner tall columnar cell layer and outer cuboidal cell layer. Sometimes, comedo-like channels that extend into the epidermis are seen.^[2]^ This report describes a rare case of isolated TAA on the palm, a non-hair bearing area.

## 2 Case report

**Figure 1:**
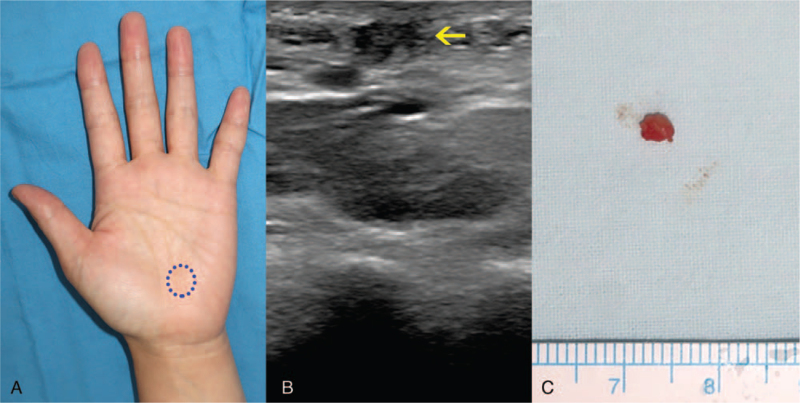
Preoperative findings and excised surgical specimen. (A) A well-circumscribed nodule on the proximal palm (blue circle). (B) Ultrasonography of the patient's left palm shows 0.4 cm sized hypoechoic nodule (yellow arrow). (C) Excised mass shows a solid nodule measuring about 0.4 cm.

**Figure 2:**
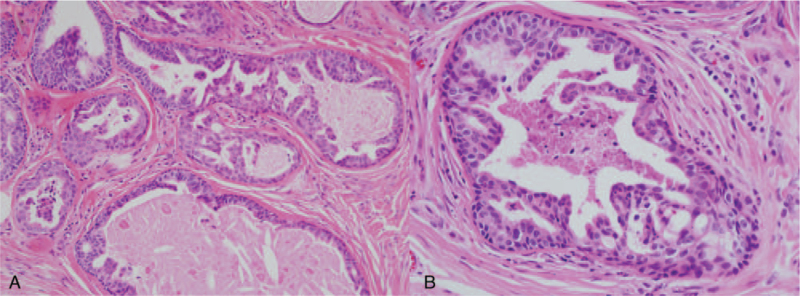
Histologic findings. (A) Well-differentiated tubular structures in the dermis, separated by a scanty hyaline stroma (×200, H&E stain). (B) Double lining of apocrine cells composed of inner tall columnar cell and outer cuboidal cell. Decapitation secretion with luminal keratin is also seen. (×400, H&E stain).

**Figure 3:**
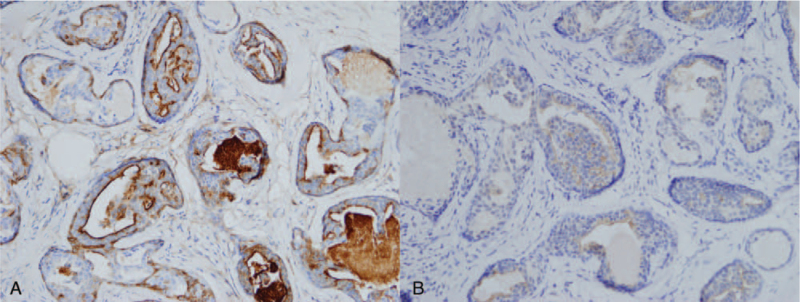
Immunohistochemical findings. (A) Positive stain for epithelial membrane antigen on luminal cells. (×200, EMA stain). (B) Positive cytoplasmic stain for GCDFP-15. (×200, GCDFP-15 stain). EMA = epithelial membrane antigen, GCDFP-15 = gross cystic disease fluid protein-15.

A 40-year-old woman presented to our department with an incidental hand mass, which was noticed 3 years ago. Gross physical examination revealed about 0.5-cm-sized, single, nodular, skin-colored, firm, and non-tender mass palpable over the proximal portion of her left palm (Fig. 1A). Associated regional lymphadenopathy was not found. There was no previous history of trauma or disease to her hand. Her medical history, as well as occupational history related to manual labor, was unremarkable. An imaging study was done under the impressions such as epidermal cyst, foreign body granuloma, or pilomatricoma. Ultrasonography revealed a 0.4-cm-sized hypoechoic nodule in the subcutaneous plane of the left palm at the fourth carpometacarpal joint level (Fig. 1B). Complete excision along the subcutaneous plane was performed (Fig. 1C). Histopathological examination revealed lobules of well-differentiated dilated tubular structures at the dermis level (Fig. 2A). On high power magnification, lobular pattern of apocrine structures surrounded by hyaline stroma was found. Decapitation secretion with intraluminal keratin was also found (Fig. 2B). Immunohistochemistry for epithelial membrane antigen was positive in the lining cells of the tubular structures (Fig. 3A). Diffuse cytoplasmic stain was evident for gross cystic disease fluid protein-15 immunohistochemistry (Fig. 3B). Based on these histology findings, the diagnosis of TAA was suggested. The wound after excision healed uneventfully. She is being followed up for 1 year without signs of recurrence.

## 3 Discussion

According to its cell type, adnexal tumors can be classified into 4 types, namely apocrine, eccrine, follicular, and sebaceous. Tubular apocrine adenoma (TAA) is a rare sweat gland neoplasm among many benign adnexal tumors. Despite its rarity, WHO classification of skin tumors dictate TAA as a distinct entity subcategorized under apocrine gland tumors.^[3]^ This entity is a synonymous term with apocrine adenoma, tubulopapillary hidradenoma, and papillary tubular adenoma in previous literatures.

The occurrence of TAA predominates in women with ratio of 2:1. Age distribution of TAA is very wide, ranging from 18 to 78 years.^[4]^ Since apocrine gland develop in association with hair follicle, TAA is usually not expected to arise in a hairless, and eccrine-dominant region of the body. The most common site of involvement is the scalp, followed by less common locations such as axilla and anogenital area.^[2]^ These locations coincide with usual distribution of the apocrine gland, which is feasible considering its histological origin. The palm, as in our case, is the last anatomic region to expect the occurrence of TAA since the thick palmar skin lack any skin appendages except eccrine gland. As of 2021, PubMed search for this entity reveals only about 150 reports, most of which are TAA of the scalp and anogenital area. TAA in some uncommon locations such as the vulva and eyelid have been reported.^[5,6]^ However, to our knowledge, this case is the first report to document an ectopic presentation in the palmar skin.

Histologic features of TAA is characterized by lobules of well-differentiated tubular structures mainly at the dermis, and sometimes extending to the subcutis.^[4]^ The tubule is lined with 2 rows of epithelial cells which show decapitation secretion. The lining cells are cuboidal to columnar, with eosinophilic cytoplasm and round nuclei.^[7]^ On immunohistochemistry, TAA is positive for epithelial membrane antigen, gross cystic disease fluid protein-15. TAA needs differentiation from syringocystadenoma papilliferum, which show dilated cystic invaginations and papillary projections. These features are not evident in TAA.

Generally, TAA is considered a benign disease. However, it may act in a locally aggressive pattern. Luminal cell bridging, calcification, cellular atypia, and perineural invasion suggest the need for a closer follow-up.^[6]^

## Author contributions

**Data curation:** Jeong Hwa Seo, Jong Yun Choi.

**Formal analysis:** Bommie Florence Seo.

**Investigation:** Jeong Hwa Seo.

**Pathology review:** Jangyoun Choi, Jong Yun Choi.

**Resources:** Bommie Florence Seo, Jeong Hwa Seo, Sung-No Jung, Jangyoun Choi.

**Supervision:** Ho Kwon.

**Visualization:** Jong Yun Choi.

**Writing – original draft:** Jangyoun Choi.

**Writing – review & editing:** Sung-No Jung, Bommie Florence Seo, Ho Kwon.
